# Diamond step-index nanowaveguide to structure light efficiently in near and deep ultraviolet regimes

**DOI:** 10.1038/s41598-020-75718-x

**Published:** 2020-10-28

**Authors:** Nasir Mahmood, Muhammad Qasim Mehmood, Farooq Ahmad Tahir

**Affiliations:** 1 Research Institute for Microwave and Millimeter-Wave Studies (RIMMS), National University of Sciences and Technology (NUST), Islamabad, 46000 Pakistan; 2 NanoTech Lab, Department of Electrical Engineering, Information Technology University (ITU) of the Punjab, Ferozepur Road, Lahore, 54600 Pakistan

**Keywords:** Nanoscience and technology, Nanoscale devices, Nanoscale materials

## Abstract

Two-dimensional metamaterials, consisting of an array of ultrathin building blocks, offer a versatile and compact platform for tailoring the properties of the electromagnetic waves. Such flat metasurfaces provide a unique solution to circumvent the limitations imposed by their three-dimensional counterparts. Albeit several successful demonstrations of metasurfaces have been presented in the visible, infrared, and terahertz regimes, etc., there is hardly any demonstration for ultraviolet wavelengths due to the unavailability of the appropriate lossless materials. Here, we present diamond as an ultra-low loss material for the near and deep ultraviolet (UV) light and engineer diamond step-index nanowaveguides (DSINs) to achieve full control over the phase and amplitude of the incident wave. A comprehensive analytical solution of step-index nanowaveguides (supported by the numerical study) is provided to describe the underlying mechanism of such controlled wavefront shaping. Due to the ultra-low loss nature of diamond in near and deep UV regimes, our DSINs and metasurfaces designed (from them) exhibit a decent efficiency of ≈ 84% over the entire spectrum of interest. To verify this high efficiency and absolute control over wavefront, we have designed polarization-insensitive meta-holograms through optimized DSINs for operational wavelength λ = 250 nm.

## Introduction

Shorter wavelengths such as near and deep ultraviolet (UV) light promise tremendous potential for several applications like sensing, photolithography and high-resolution imaging. Artificially engineered materials can promise the realization of such compact solutions at these shorter wavelengths. For about two decades, artificially engineered photonic metamaterials attracted wide attention and have been extensity studied by diverse research communities due to the possession of unprecedented capabilities of tailoring the propagation of electromagnetic waves^1,2^. Due to engineered permittivity and permeability of the photonic metamaterials and their strong interaction with the incident electromagnetic waves, these metamaterials present significantly enhanced capabilities in visible and near-infrared regime^3–5^, which may not be conceivable through naturally existing materials^6^. However, strong dispersion, three dimensional (3D) bulky structure, fabrication challenges (specifically precise multilayer stacking) at optical frequencies and significant loss has considerably degraded the performance of metamaterials-based photonic devices^7–9^. These listed limitations have hampered the metamaterial’s integration in miniaturized on-chip practical applications also with fast-growing state-of-the-art complementary metal-oxide-semiconductor (CMOS) technologies^7,9^. Recently, the advent of the two dimensional (2D) version of bulky metamaterials dubbed as metasurfaces^10,11^, have revolutionized the field of electromagnetic waves manipulation. These metasurfaces comprise a 2D array of spatially varying subwavelength thick plasmonic or dielectric meta-atoms, enabling us to tailor the wavefront of incident electromagnetic waves by manipulating their amplitude, phase, and polarization properties. Therefore, any arbitrary phase distribution for wavefront manipulation can be implemented by spatially varying the geometric parameters (size, shape, and orientation) of the optimized meat-atoms. Along with other versatile applications such as flat lenses^12–14^, broadband wave-plates^15,16^, perfect absorber^17^ and optical vortex beam generation^18–22^, metasurfaces provide a unique solution and excellent flexibility in the implementation and realization of computer-generated holograms^23–25^.

Metasurfaces consisting of metallic materials are successfully demonstrated for the wavelengths ranging from terahertz (THz) to near-infrared (NIR) domains^26–28^. These plasmonic metasurfaces suffer from temperature instability, intrinsic Ohmic losses, chemical inertness and non-compatibility with CMOS technologies^29^. Above mentioned limitations substantially deteriorate their performance for visible to ultraviolet spectrums, where a diverse range of practical applications is of interest. For shorter wavelengths, however, Aluminum (Al) possesses potentially better response^30^, again induced oxidation and complex fabrication requirements while scaling down have degraded their overall performance^31^. Moreover, the absence of magnetic dipole resonance in transmission-type plasmonic metasurfaces has significantly depreciated their overall transmission efficiency and restricted it to 25% in the visible regime^32^. In parallel, high-index dielectric materials possessing a transparent window $\left( {k \approx 0} \right)$ in the region of interest and excitation of both (electric and magnetic dipole) resonances have dominant advantages over these plasmonic counterparts for a wide range of optical wavelengths. Based upon the concept of index waveguide theory or Mie resonances, these all-dielectric metasurfaces appearing as a best-suited candidate for the realization of transmission-type efficient solutions. In this regard, lossless dielectric materials like gallium nitride (GaN), titanium dioxide (TiO_2_), silicon nitride (Si_3_N_4_) and hydrogenated amorphous silicon (a-Si:H) present themselves as ideal contestants and successfully employed to realize numerous applications in infrared and visible spectrums^33–36^. However, these dielectric materials offer significant absorption for the near and deep ultraviolet regime, also sophisticated fabrication techniques required to implement these metasurfaces have hampered their integration with practical applications. So, the hunt for an appropriate material which ensures an efficient and miniaturized solution in the near and deep ultraviolet regimes is going on.

**Figure 1:**
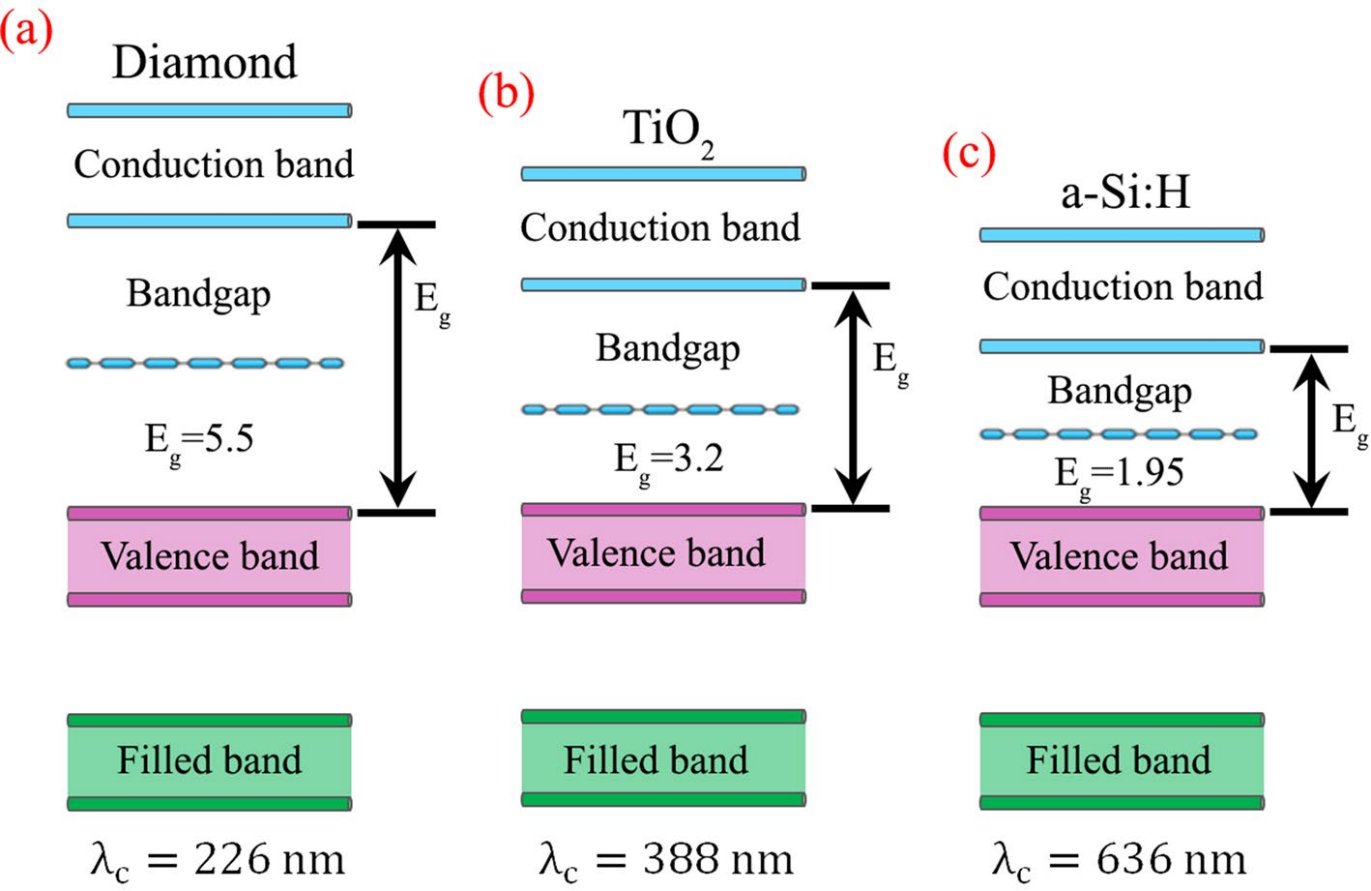
Bandgap investigation. (**a**) Diamond, having a bandgap of 5.5 with $\lambda_{{\text{c}}} = 226\;{\text{nm}}$, (**b**) Titanium dioxide (TiO_2_), having a bandgap of 3.2 with $\lambda_{{\text{c}}} = 338\;{\text{nm}}$, (**c**) Hydrogenated amorphous silicon (a-Si:H), having a bandgap of 1.95 with $\lambda_{{\text{c}}} = 636\;{\text{nm}}$. All the sub-figures are created using PowerPoint software from Microsoft 2020^45^.

To find an appropriate material with a transparency window in the desired spectral band, Fig. 1 illustartes the energy bandgap characteristics of three different dielectric materials diamond, titanium dioxide, and hydrogenated amorphous silicon (a-Si:H), respectively. With the help of bandgap energy, cutoff wavelength for any material can be calculated using $${\uplambda }_{{\text{c}}} = {\raise0.7ex\hbox{${h*c}$} \!\mathord{\left/ {\vphantom {{h*c} {E_{g} }}}\right.\kern-\nulldelimiterspace} \!\lower0.7ex\hbox{${E_{g} }$}},$$ where *h* is the Planck’s constant, ‘*c*’ represents the speed of light and *E*_*g*_ is the bandgap energy. The transparency window can be defined as the range of wavelengths for which the extinction coefficient of the materials is zero $$\left( {k \approx 0} \right)$$ and it contains all the values greater than the cutoff wavelength (λ_c_). Figure 1 predicts that as compared to the other two dielectric materials (TiO_2_ and a-Si:H) diamond exhibits a smaller cutoff wavelength $$\left( {\lambda_{{\text{c}}} = 226\;{\text{nm}}} \right)$$ which validates its applicability for the near and deep ultraviolet wavelengths. This specific behavior of diamond material is also validated from its optical characteristics described in Fig. 3a.

Most of the other dielectric materials (e.g., ${\text{CaF}}_{2}$, ${\text{SiO}}_{2} ,$ and ${\text{MgF}}_{2}$) possessing a transparent window $\left( {k \approx 0} \right)$ for ultraviolet wavelengths, have a lower index of refraction which requires costly and challenging fabrication techniques. Low refractive index materials are not difficult to fabricate themselves but it is due to the fact that the unit cell made of these materials, requires a larger height-to-diameter ratio (aspect ratio) for the realization of complete (0–2π) phase control. As the refractive index of dielectric material increases, for complete phase coverage, the required height of the unit cell decrease, eventually resulting in a lower aspect ratio which facilitates the fabrication and vice versa. This point can also be understood with the help of the following mathematical calculation:

$$\begin{aligned} & n_{{{\text{eff}}}} = 1 \to 1.5\quad {\it\text{H}}_{1} = 1000 \;{\text{nm}}\quad n_{{{\text{eff}}}} = 1 \to 3.25\quad {\it\text{H}}_{2} = 400\;{\text{nm}} \\ & \phi_{1} = \frac{2\pi }{{633\;{\text{nm}}}} \cdot 1 \cdot 1000\;{\text{nm}}\quad \phi_{1} = 568^{ \circ } \quad \phi_{4} = \frac{2\pi }{{633\;{\text{nm}}}} \cdot 1 \cdot 400\;{\text{nm}}\quad \phi_{4} = 227^{ \circ } \\ & \phi_{2} = \frac{2\pi }{{633\;{\text{nm}}}} \cdot 1.5 \cdot 1000\;{\text{nm}}\quad \phi_{2} = 853^{ \circ } \quad \phi_{5} = \frac{2\pi }{{633\;{\text{nm}}}} \cdot 3.25 \cdot 400\;{\text{nm}}\quad \phi_{5} = 740^{ \circ } \\ & \Delta \phi_{3} = \phi_{2} - \phi_{1} ,\;\; \Delta \phi_{3} = 853^{ \circ } - 568^{ \circ } = 285^{ \circ } \quad \Delta \phi_{6} = \phi_{5} - \phi_{4} ,\;\; \Delta \phi_{6} = 740^{ \circ } - 227^{ \circ } = 513^{ \circ } \\ \end{aligned}$$

Here $\left( {\phi = \frac{2\pi }{{\lambda_{{\text{d}}} }} \cdot n_{{{\text{eff}}}} \cdot {\it\text{H}}} \right)$ is used to calculate the propagating phase where ‘n_eff_’ is the effective refractive index ‘H’ is the thickness of the material and ‘$\lambda_{{\text{d}}}$’ is the design wavelength. In the above calculation, two dielectric materials with different thicknesses and refractive indices ($n = 1.5,\;{\it\text{H}}_{1} = 1000\;{\text{nm}}$, and $n = 3.25,\;{\it\text{H}}_{2} = 400\;{\text{nm}}$) are chosen for the clarity of the concept. It is obvious from the above calculation that a dielectric material with a lower refractive index $\left( {n = 1.5} \right)$ is unable to control complete $(360^{{\text{o}}} )$ phase profile even with a height of 1000 nm whereas other dielectric material with high refractive index $\left( {n = 3.25} \right)$ can easily accumulate complete phase profile with height of 400 nm. It is concluded that to ensure miniaturized on-chip devices, dielectric materials with a high refractive index would be an ideal candidate. A detailed analysis of few other dielectric materials is presented in Table 1 which shows that diamond and silicon nitride (Si_3_N_4_) possessing a transparency window for the desired spectral band while others not, however, Si_3_N_4_ possesses a lower index of refraction as compared to diamond.

**Table 1:** Material comparison^18,37–42^.

Parameter name	Silicon dioxide	Diamond	Silicon nitride	Gallium nitride	Titanium dioxide	a-Si:H	Amorphous silicon
Bandgap	9.0	5.5	5.0	3.4	3.2	1.95	1.75
Cutoff wavelength	138 nm	226 nm	248 nm	365 nm	388 nm	636 nm	709 nm
Operational wavelength	633 nm	700 nm	633 nm	633 nm	660 nm	633 nm	633 nm
Nano-antenna height	–	1000 nm	695 nm	600 nm	600 nm	400	370 nm
Optical constant	1.450	2.406	2.039	2.3848	2.586	3.2495	4.22
	0.00	0.00	0.00	0.00	0.00	0.047	0.47
Aspect ratio	–	10.0	7.0	8.57	7.06	4.0	3.7
Period of unit cell	–	300 nm	416 nm	330 nm	300 nm	300 nm	350 nm

All the parts of the table are created using PowerPoint software from Microsoft 2020^45^.

The idea of using diamond as an efficient dielectric material for metasurfaces working in the ultraviolet regime has been proposed and few research papers are reported^38,43^. Hu, J. et al*.* reported full-field simulations-based high-quality factor diamond metasurfaces that enhance optical chirality by over three orders of magnitude in the ultraviolet regime^38^. The diamond nanostructures enable ultraviolet Mie resonance while the asymmetry in adjacent disk lattice activates high-quality factor resonances that significantly enhance the circular dichroism (CD) and increase the electromagnetic field intensities. This research work utilized 60 nm thick diamond nanopillar as a fundamental building block. Spectral overlapping of the dipole modes exhibits a Kerker like condition where the transmission approaches unity. This research work presents a totally different phenomenon from our proposed phase-dictated device in which complete (0–2π) phase control is acquired by spatially varying the physical dimension of the unit cell. In another research work, Huang, T. et al*.* leverages diamond as a high refractive index ($n \sim 2.4$ at visible wavelengths) material to design and fabricate high-numerical-aperture all-diamond immersion metalens. A fundamental building block consisting a 1 µm thick diamond pillar extending from the surface of the single-crystal diamond is chosen to build the metasurface. The fundamental building block is optimized and the metasurface is designed for an operational wavelength of $\lambda_{{\text{d}}} = 700\;{\text{nm}}$.

Here, we utilize diamond as an ideal dielectric material, for the near and deep ultraviolet light, to realize highly efficient phenomena through artificially engineering it (diamond). Its sufficient high refractive index and absolute transparent window for our desired range of wavelengths make it a best-suited candidate for the realization of compact devices. From Fig. 1, it is safely stated that our proposed diamond material is the best-suited candidate for the implementation of all-dielectric metasurfaces in the near and deep ultraviolet regimes with sufficient high index of refraction and absolute zero extinction coefficient. We propose diamond step-index nanowaveguides (DSINs) to efficiently control the phase and amplitude of the incident UV light desirably. Theoretical modeling of DSINs is presented in detail and the underlying mechanism to control the phase through varying the diameter (of DSINs) is provided comprehensively. Numerical optimization is performed to validate the proposed theoretical modeling which enables complete (0–2π) phase coverage (with maximum possible transmission amplitude) by spatially varying the diameter of DSINs. To verify this absolute control over amplitude and phase of the incident light’s wavefronts, the optimized DSINs are used to implement the polarization-insensitive meta-holograms for near and deep UV regimes (particularly for an operational wavelength of λ = 250 nm). As compared to existing dielectric materials (${\text{TiO}}_{2}$, ${\text{Si}}_{3} {\text{N}}_{4} , {\text{GaN}}\;{\text{and}}$ a-Si:H), diamond exhibits very high efficiency for the wavelengths of interest while its sufficiently high refractive index keeps the aspect ratio (${\text{AR}} = 5.7$ for our case) within the fabricate-able limits. Our meta-hologram exhibits the words “NUST” and “ITU” for wavelengths λ = 250 nm which illustrate sufficient high transmission efficiency and image fidelity. This demonstration will surely open an avenue to explore diamond-based metamaterials and investigate possibilities of their fabrications at the nanoscale.

## Theoretical modelling of DSINs and their underlying mechanism for absolute wavefront engineering

**Figure 2:**
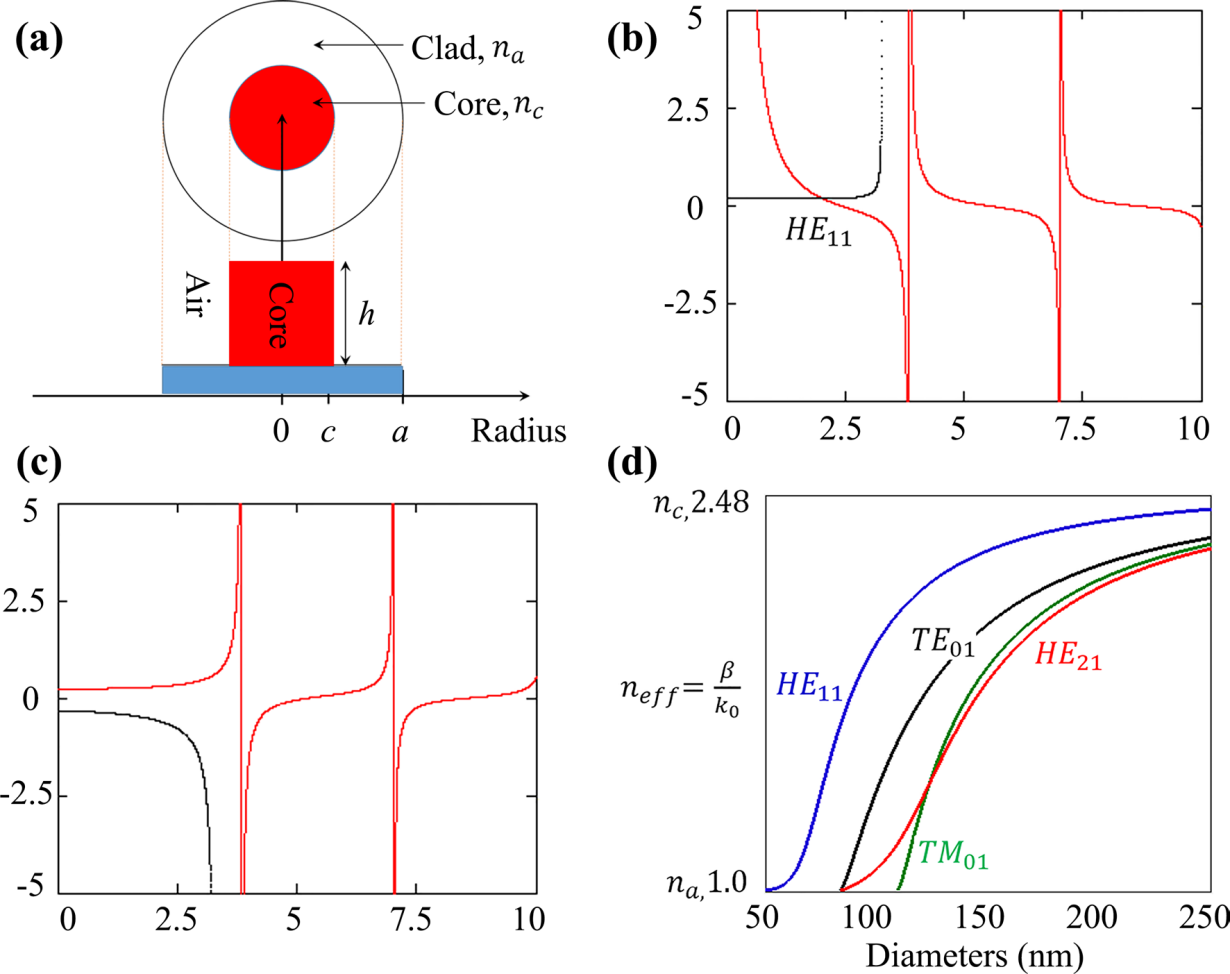
Indexed waveguide theory. (**a**) Geometry and index profile of a circular step-indexed waveguide, where $n_{c}$ and $n_{a}$ indicate the refractive indices while “$c$” and “$a$” represent the radii of core (diamond) and cladding (air), respectively. (**b**) Graphic prediction of the propagation constant of HE modes considering $n_{c} = 2.48$, $n_{a} = 1,\; a = 80\;{\text{nm}}$ and $\lambda = 350\;{\text{nm}}$ which results in normalized frequency $W = k_{0} a\sqrt {\left( {n_{c}^{2} - n_{a}^{2} } \right)}$ as 3.259. (**c**) Graphic prediction of the propagation constant of EH modes considering $n_{c} = 2.48$, $n_{a} = 1, \;a = 80\;{\text{nm }}$ and $\lambda = 350\;{\text{nm}}$ which results in normalized frequency $W = k_{0} a\sqrt {\left( {n_{c}^{2} - n_{a}^{2} } \right)}$ as 3.259. (**d**) Representation of effective refractive index as a function of the diameter of nanopillar for some lower order modes of a step-index waveguide. All the sub-figure are created using PowerPoint software from Microsoft 2020^38^.

The ultimate goal is to obtain full control over the wavefronts of UV light through spatially varying the diameter of meta-atoms. Any phase-dictated phenomena can be implemented if such dynamic wavefront control is available. Our proposed DSINs can provide such control for the UV light. To understand the underlying physics of this control, we comprehensively developed the theoretical modeling for our DSINs. Figure 2a shows the structure and index profile of the step-index waveguide where the core is characterized as DSIN, “*a*” and “*c”* represent the radii while $n_{{\text{a}}}$ and $n_{{\text{c}}}$ indicate the refractive indices of cladding (air) and core (diamond), respectively. Strong confinement of propagating modes within the waveguide is ensured by the significant difference of the refractive index of the DSIN and surrounding medium (air). Standard Maxwell’s equations can be helpful in determining the transverse and longitudinal components of the electromagnetic field within the DSIN. Detailed derivation can be found in^18,37^. According to the indexed waveguide theory, by adjusting the dimension (diameter in our case) of the index waveguide, the effective refractive index of the propagating mode can be modified, which can also be verified from the mathematical derivations provided below. At the core clad interface (*r* = *a*), boundary conditions must be satisfied and continuity of $E_{\phi } , E_{z} , H_{\phi } , \;{\text{and}}\;H_{z}$ field components yield the following relationship

1 $$SJ_{\ell } \left( {fa} \right) - UK_{\ell } \left( {ga} \right) = 0$$

2 $$S\left( {\frac{i\ell }{{f^{2} a}}J_{\ell } \left( {fa} \right)} \right) + T\left( { - \frac{\omega \mu }{{f\beta }}J_{\ell }^{^{\prime}} \left( {fa} \right)} \right) + U\left( {\frac{i\ell }{{g^{2} a}}K_{\ell } \left( {ga} \right)} \right) + V\left( { - \frac{\omega \mu }{{g\beta }}K_{\ell }^{^{\prime}} \left( {ga} \right)} \right) = 0$$

3 $$TJ_{\ell } \left( {fa} \right) - VK_{\ell } \left( {ga} \right) = 0$$

4 $$S\left( {\frac{{\omega \varepsilon_{1} }}{f\beta }J_{\ell }^{^{\prime}} \left( {fa} \right)} \right) + T\left( {\frac{i\ell }{{f^{2} a}}J_{\ell } \left( {fa} \right)} \right) + U\left( {\frac{{\omega \varepsilon_{2} }}{g\beta }K_{\ell }^{^{\prime}} \left( {ga} \right)} \right) + V\left( {\frac{i\ell }{{g^{2} a}}K_{\ell } \left( {ga} \right)} \right) = 0$$

where *S*, *T*, *U* and *V* are arbitrary constants. The relationship of parameters $f$ and $g$ with the propagation constant $\beta$ is defined as $f = \left[ {\left( {\frac{{n_{{\text{c}}} \omega }}{c}} \right)^{2} - \beta^{2} } \right]^{1/2}$, $g = \left[ {\beta^{2} - \left( {\frac{{n_{{\text{a}}} \omega }}{c}} \right)^{2} } \right]^{1/2}$ and $J_{\ell }^{^{\prime}} \left( {fr} \right) = dJ_{\ell } \left( {fr} \right)/d\left( {fr} \right)$, $K_{\ell }^{^{\prime}} \left( {gr} \right) = dK_{\ell } \left( {gr} \right)/d\left( {gr} \right)$, $\varepsilon_{1} = \varepsilon_{0} n_{{\text{c}}}^{2}$ and $\varepsilon_{2} = \varepsilon_{0} n_{{\text{a}}}^{2}$, where $J$ and $K$ represent Bessel and modified Bessel Functions respectively while $\ell$ can be defined in a way similar to the topological charge of the optical vortex. A nontrivial solution of Eqs. (1–4) can be determined for *S*, *T*, *U* and *V*, provided the determinants of their coefficients vanishes. By enforcing the above condition, the following mode matching equation can be achieved. This relationship will help us in achieving “$\beta$,” the unknown propagation constant.

5 $$\left( {\frac{{J_{\ell }^{^{\prime}} \left( {fa} \right)}}{{faJ_{\ell } \left( {fa} \right)}} + \frac{{K_{\ell }^{^{\prime}} \left( {ga} \right)}}{{gaK_{\ell } \left( {ga} \right)}}} \right)\left( {\frac{{n_{c}^{2} J_{\ell }^{^{\prime}} \left( {fa} \right)}}{{faJ_{\ell } \left( {fa} \right)}} + \frac{{n_{a}^{2} K_{\ell }^{^{\prime}} \left( {ga} \right)}}{{gaK_{\ell } \left( {ga} \right)}}} \right) = \ell^{2} \left[ {\left( \frac{1}{ga} \right)^{2} + \left( \frac{1}{fa} \right)^{2} } \right]^{2} \left( {\frac{\beta }{{k_{0} }}} \right)^{2}$$

Here “$\beta$” is unknown and $k_{0}$ is a free space wavenumber. In Eq. (5), the parameters $f$, $g$ and $\beta$ are intermixed, forming a transcendental equation. Due to the quadratic nature of Eq. (5), two different types of solutions are possible. By considering the most general case, when $\ell \ne 0$, all six electromagnetic field components will be present (non-vanishing), conventionally designating these modes as hybrid (HE and EH) modes^37^. These solutions can be represented as.

For HE modes

6 $$\frac{{J_{\ell - 1} \left( {fa} \right)}}{{faJ_{\ell } \left( {fa} \right)}} = - \left( {\frac{{n_{{\text{c}}}^{2} + n_{{\text{a}}}^{2} }}{{2n_{{\text{c}}}^{2} }}} \right)\frac{{K_{\ell }^{^{\prime}} \left( {ga} \right)}}{{gaK_{\ell } \left( {ga} \right)}} + \left( {\frac{1}{{\left( {fa} \right)^{2} }} - R} \right)$$

For EH modes:

7 $$\frac{{J_{\ell + 1} \left( {fa} \right)}}{{faJ_{\ell } \left( {fa} \right)}} = \left( {\frac{{n_{{\text{c}}}^{2} + n_{{\text{a}}}^{2} }}{{2n_{{\text{c}}}^{2} }}} \right)\frac{{K_{\ell }^{^{\prime}} \left( {ga} \right)}}{{gaK_{\ell } \left( {ga} \right)}} + \left( {\frac{1}{{\left( {fa} \right)^{2} }} - R} \right)$$

where

8 $$R = \left( {\left( {\frac{{n_{{\text{c}}}^{2} - n_{{\text{a}}}^{2} }}{{2n_{{\text{c}}}^{2} }}} \right)^{2} \left( {\frac{{K_{\ell }^{^{\prime}} \left( {ga} \right)}}{{gaK_{\ell } \left( {ga} \right)}}} \right)^{2} + \left( {\frac{\ell \beta }{{n_{{\text{c}}} k_{0} }}} \right)^{2} \left( {\frac{1}{{g^{2} a^{2} }} + \frac{1}{{f^{2} a^{2} }}} \right)^{2} } \right)^{1/2}$$

Above mention Eqs. (6–7) can be solved graphically where each side of the equation can be plotted as a function of *fa*. Here we consider $\left( {ga} \right)^{2} = \left( {n_{{\text{c}}}^{2} - n_{{\text{a}}}^{2} } \right)k_{0}^{2} - \left( {fa} \right)^{2}$.

For $$\ell = 1,$$ graphical determination of the propagation constants for HE and EH modes are expressed in Fig. 2b,c, which indicates the two curves representing the two sides for HE and EH mode condition equations. Here for a particular case, we consider $$n_{{\text{c}}} = 2.48$$,$$n_{{\text{a}}} = 1,\;a = 80\;{\text{nm}}$$ and $$\lambda = 350\;{\text{nm}}$$ which results in normalized frequency $$W = k_{0} a\sqrt {\left( {n_{{\text{c}}}^{2} - n_{{\text{a}}}^{2} } \right)}$$ as 3.259. Figure 2b shows that, for the above-listed values, only fundamental mode (HE_11_) is propagating as there is only one intersection available. It is also evident from Fig. 2b that HE_11_ mode has no cut off wavelength; in other words, this mode always be propagating regardless of the value of normalized frequency *W*. Similarly, Fig. 2c indicates that for these particular values, there is no EH propagating mode because there is no intersection point of two curves is available. On the same lines, as the dimension of indexed waveguide increases, additional higher-order modes start appearing and this phenomenon is depicted in Fig. 2d where normalized propagation constant is plotted vs. diameter of the nanopillar. All other higher-order EH_1m_ and HE_1m_ modes have specific cutoff values but fundamental mode HE_11_ doesn’t have any cutoff value. The cutoff values for other higher-order EH_1m_ and HE_1m_ modes for $${\raise0.7ex\hbox{$a$} \!\mathord{\left/ {\vphantom {a \lambda }}\right.\kern-\nulldelimiterspace} \!\lower0.7ex\hbox{$\lambda $}}$$ given by.

For EH modes

9 $$\left( {\frac{a}{\lambda }} \right)_{{1{\text{m}}}} = \frac{3.832}{{2\pi \left( {n_{{\text{c}}}^{2} - n_{{\text{a}}}^{2} } \right)^{1/2} }},\;\;\frac{7.016}{{2\pi \left( {n_{{\text{c}}}^{2} - n_{{\text{a}}}^{2} } \right)^{1/2} }},\;\;\frac{10.173}{{2\pi \left( {n_{{\text{c}}}^{2} - n_{{\text{a}}}^{2} } \right)^{1/2} }} \cdots$$

For HE modes

10 $$\left( {\frac{a}{\lambda }} \right)_{{1{\text{m}}}} = 0,\;\; \frac{3.832}{{2\pi \left( {n_{{\text{c}}}^{2} - n_{{\text{a}}}^{2} } \right)^{1/2} }},\;\;\frac{7.016}{{2\pi \left( {n_{{\text{c}}}^{2} - n_{{\text{a}}}^{2} } \right)^{1/2} }}, \cdots$$

Presence of both $E_{z}$ and $H_{z}$ components validate the existence or propagation of hybrid modes. The designation of HE and EH modes is purely based on their relative contribution to a transverse component of the electromagnetic field at a particular reference point. A propagating mode is designated as ${\text{HE}}_{{\ell {\text{m}}}}$ mode if $H_{z}$ plays a significant role and vice versa. Propagation constant *β* is an essential characteristic of any propagating mode, which is eventually a function of normalized frequency *W* (or frequency ω). Figure 2d shows the behavior of the mode index of the confined mode as a function of the diameter of the DSIN. The relationship between the mode index and phase constant of propagating mode can be described as

11 $$n_{{{\text{eff}}}} = \frac{\beta }{{k_{0} }}$$

Here $n_{{{\text{eff}}}}$ is called an effective refractive index of confined mode which varies between $n_{{\text{c}}}$ and $n_{{\text{a}}}$. Subsequently, according to indexed waveguide theory, the phase imparted by each DSIN of specific diameter can be calculated in terms of effective refractive index as:

12 $${\phi = \frac{2\pi }{{\lambda_{{\text{d}}} }} \cdot n_{{{\text{eff}}}} \cdot {\it\text{H}}}$$

where $\lambda_{d}$ is the design wavelength.

## DSINs based highly efficient near and deep UV meta-holograms

**Figure 3:**
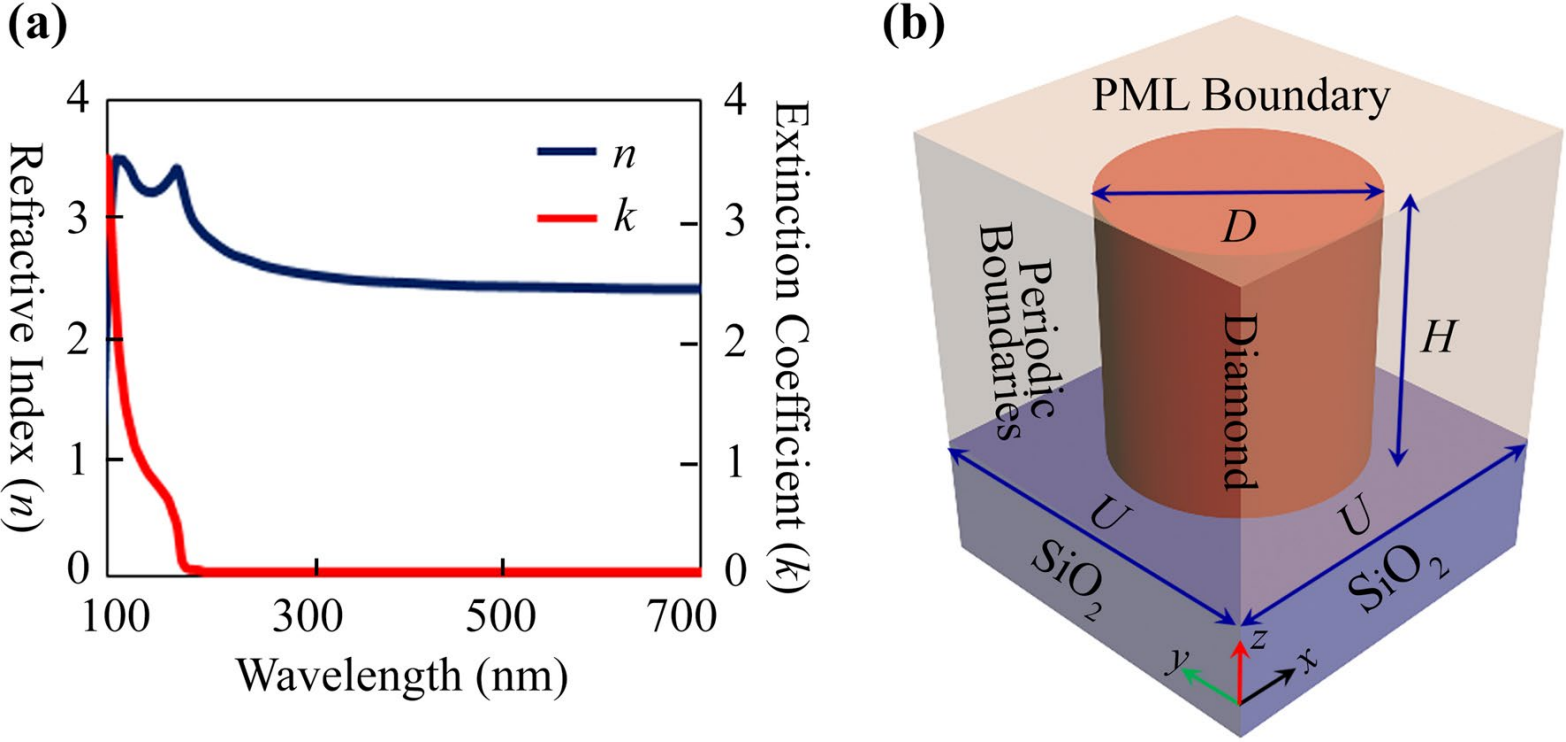
Optical properties of diamond material and schematic of diamond step-index nanowaveguide (DSIN) along with simulation boundary conditions. (**a**) Real (blue) and imaginary (red) part of the complex refractive index of diamond material for the wavelength ranging from 100 to 700 nm. (**b**) Side view of the DSIN along with boundary conditions opted for numerical simulations, showing its diameter *D* and height *H* while *U* represents the periodicity of the unit cell. This configuration is numerically simulated using the Finite-Difference Time-Domain technique with periodic boundaries along *x*-, *y*-axis and perfect matching layer (PML) boundaries along the *z*-axis. All the figures are created using PowerPoint software from Microsoft 2020 and 3ds Max from Autodesk Inc.^38,40^.

The realization of phase-only meta-holograms requires the utilization of complete phase distribution obtained through numerical optimization of the fundamental building block (here diamond step-index nanowaveguide) in such a way to demonstrate the maximum possible transmission amplitude for desired operational wavelengths. Here in this work, DSINs with the thickness of $H = 400\;{\text{nm}}$ are numerically optimized and used as building blocks for near and deep UV wavelengths. Figure 3a describes the complex refractive index of the diamond material for wavelength ranging from 100 to 700 nm^39^, which shows that diamond possesses an absolute transparent window $\left( {k \approx 0} \right)$ for wavelengths greater than 200 nm. The choice of circular cylindrical geometry of the DSIN is due to its unique property of polarization-insensitivity. Figure 3b shows the DSIN patterned on the glass substrate where *D* and *H* represent its diameter and height, while *U* is the lattice constant of the unit cell.

**Figure 4:**
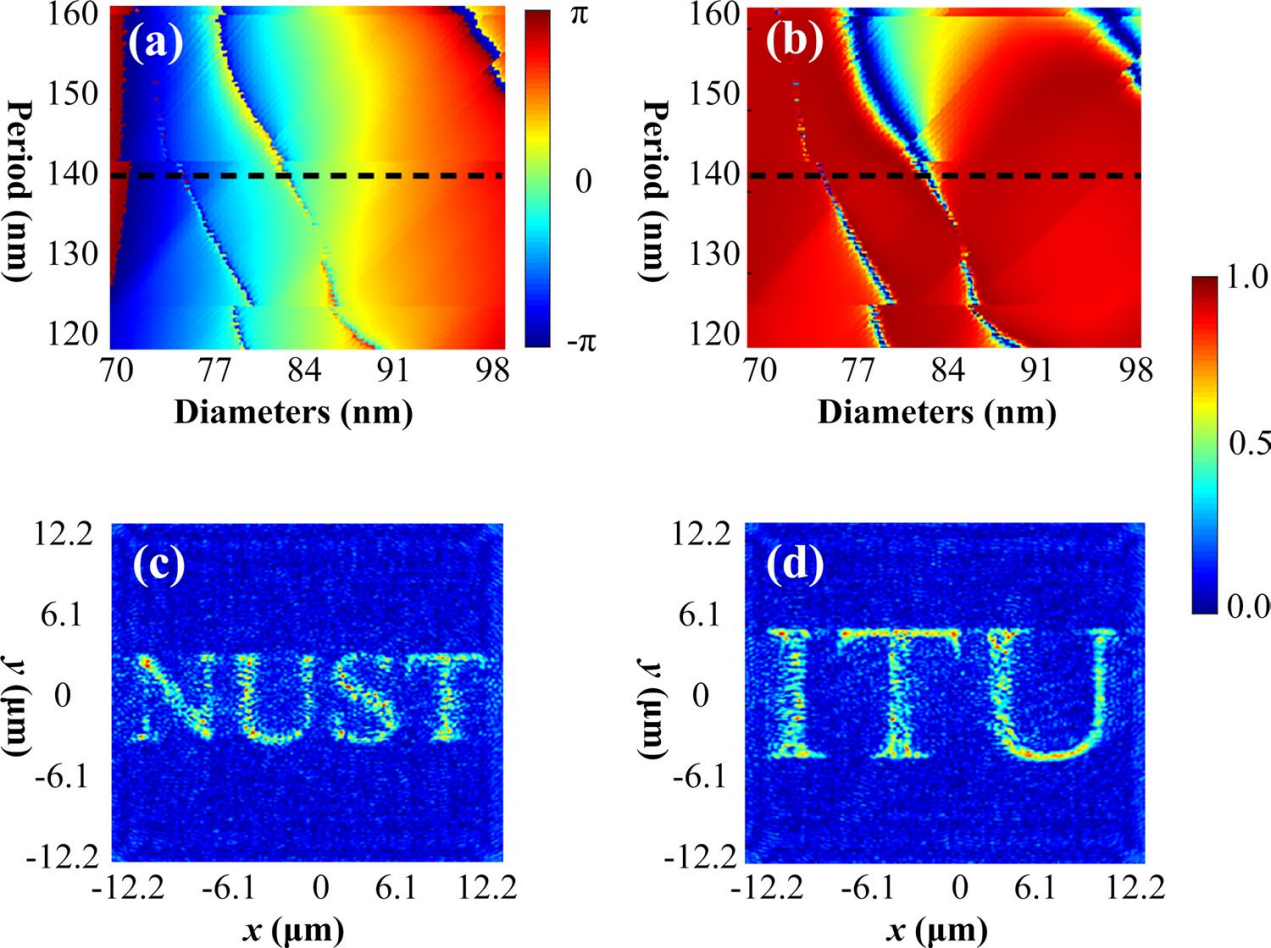
For the design wavelength $\lambda_{d} =$ 250 nm. (**a**) Surface plot of the phase profile obtained by sweeping the period from 120 to 160 nm for a range of diameter varying from 70 to 98 nm allows us to choose the optimized value of period as 140 nm. (**b**) Surface plot of transmission profile for the same range of period and diameter as mentioned for phase profile. Dashed lines indicate the optimized value of period as *U* = 140 nm achieving 94.1% average transmission efficiency. (**c**, **d**) Simulation results of designed meta-holograms, showing the holographic images as “NUST” and “ITU”. Simulated metasurfaces are 12.2 μm × 12.2 μm in size containing 175 × 175 pixels where the focal point is set as 14 μm away from metasurfaces. All the figures are created using PowerPoint software from Microsoft 2020^38^.

Although holography was invented back in 1948 by Dennis Gabor^41^, recently, it shows significant potential and successfully employed for numerous versatile practical applications like optical manipulation^42^, information encryption^43^, biological imaging^44^, data storage^45^, 3D displays^46^ and so on. Generation of the holographic image using metasurfaces essentially requires complete phase control via meta-atoms, where some iterative Fourier transform algorithm^47^ can be utilized to perform the numerical calculations to determine the amplitude and phase distribution of the diffracted light. Finally, the phase-only meta-holograms are constructed by translating the above extracted discrete phase distribution through spatial variation of geometric parameters of meta-atoms^48^. Inspired from the step-indexed waveguide concepts, for a design wavelength of 250 nm, the imparted phase profile is obtained numerically by spatially varying the DSIN and is presented as surface plot in Fig. 4a. The dashed line indicates the complete (0–2*π*) phase coverage for a range of diameter varying from 70 to 98 nm with an optimized value of period (lattice constant) as *U* = 140 nm. Figure 4b presents the numerically simulated behavior of transmission profile by varying diameter of DSIN vs. period of the unit cell. For an optimized value of period as *U* = 140 nm, a dashed line indicates the average 94.1% transmission efficiency for diameter ranging from 70 to 98 nm.

An iterative Fourier transform algorithm proposed by Gerchberg and Saxton also called as Gerchberg-Saxton algorithm^47^, is frequently used for phase retrieval to be used in the meta-holograms. For design wavelength $\lambda_{{\text{d}}} = 250\;{\text{nm}}$, GS algorithm script is provided with period *U* = 140 nm, focal length as 14 μm along with target image, which produces an array of 175 × 175 phase elements to be utilized for structuring the meta-hologram with 12.2 μm × 12.2 μm in size. Reconstructed holographic image showing images “NUST” and “ITU” at the desired focal plane is illustrated in Fig. 4c,d. Obtained results are in excellent agreement with MATLAB results validating the proposed concept.

**Figure 5:**
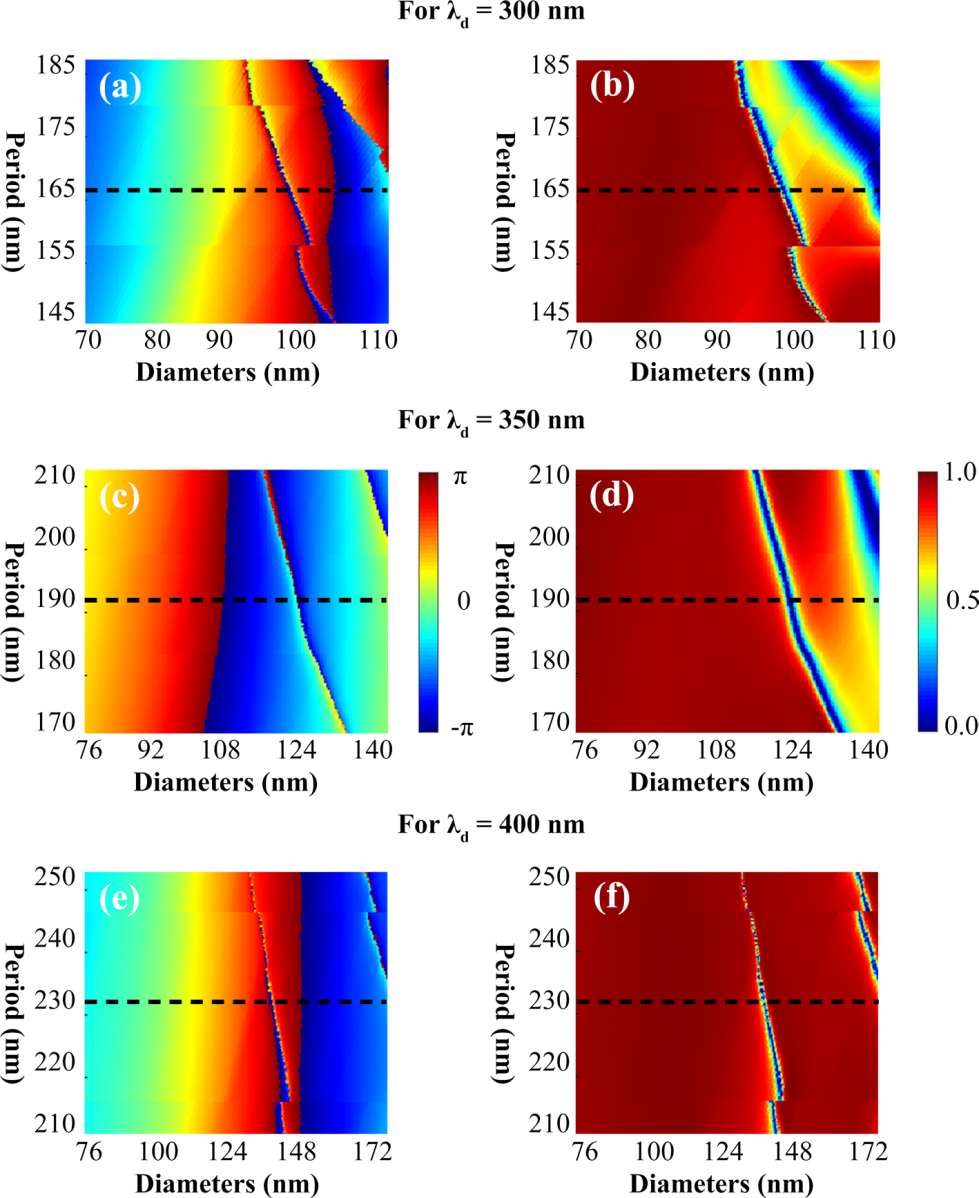
Numerical optimization for the design wavelength λ_d_ = 300, 350 and 400 nm. (**a**) For λ_d_ = 300 nm, the simulated surface plot of phase profile for a range of diameter sweeping from 70 to 110 nm with the optimized period as 165 nm. (**b**) Surface plot of the transmission spectra for a range of period vs. diameter of DSIN, as mentioned above. Dashed lines indicate the optimized value of period as *U* = 165 nm covering complete (0–2*π*) phase coverage and achieving an average 89.5% transmission efficiency. (**c**) For λ_d_ = 350 nm, the simulated surface plot of phase distribution for diameters sweeping from 76 to 140 nm where dashed line indicates the optimized period as 190 nm covering complete (0–2*π*) phase profile. (**d**) The surface profile of transmission amplitude for a range of period vs. diameter. The dashed line indicates the optimized value of periodicity as *U* = 190 nm acquiring an average 81.2% transmission efficiency. (**e**) For the design wavelength λ_d_ = 400 nm. Complete phase profile acquired numerically for a range of diameters varying from 76 to 172 nm, where the dashed line determines the optimized value of period as 230 nm. (**f**) Surface plot of and transmission spectra for a range of period vs. diameters of DSIN, as mentioned above. The dashed line indicates the optimized value of period as *U* = 230 nm achieving an average transmission efficiency as high as 96.5%. All the figures are created using PowerPoint software from Microsoft 2020^38^.

For a design wavelength of 300 nm, the surface plot of the phase profile obtained through numerical simulation by spatially varying the diameter of DSIN ranging from 70 to 110 nm is depicted in Fig. 5a. The dashed line indicates the optimized value of the period as *U* = 165 nm covering the complete (0–2*π*) phase profile. Figure 5b illustrates the numerically simulated behavior of the transmission profile by varying diameter of DSIN (ranging from 70 to 110 nm) vs. period of the unit cell achieving an average 89.5% transmission efficiency. For the design wavelength of 350 nm, the complete phase coverage is obtained through numerical simulations by spatially sweeping the diameter of DSIN and illustrated in Fig. 5c. For a range of diameter from 76 to 140 nm, complete (0–2*π*) phase distribution is achieved where the dashed line indicates the optimized value of period as *U* = 190 nm. Figure 5d presents the simulated behavior for transmission profile by varying diameter of nanopillar vs. period of the unit cell achieving an average 81.2% transmission efficiency. Finally, for a design wavelength of 400 nm, for the value of diameter ranging from 76 to 172 nm, the 2D plot of the acquired phase profile is illustrated in Fig. 5e where the dashed line indicates the optimized value of periodicity as *U* = 230 nm. Figure 5f presents the numerically simulated behavior of transmission profile by the sweeping diameter of the DSIN vs. period of the unit cell achieving an average transmission efficiency as high as 96.5%.

## Methods

Full-wave Finite-Difference Time-Domain (FDTD) simulations are performed for parametric optimization of the unit cell, whereas perfect matching layers (PML) boundary conditions are employed along the *z*-axis and periodic boundaries are employed along *x*-, *y*-axis. Here circular cylindrical diamond step-index nanowaveguides (DSINs) with a thickness of 400 nm is optimized in such a way to cover complete (0–2*π*) phase profile while maintaining maximum possible transmission amplitude for near and deep UV regimes (particularly for operational wavelengths of $\lambda =$ 250 nm, 300 nm, 350 nm and 400 nm). By changing the diameter of DSIN as a function of space, the effective refractive index of the propagating mode is altered. The effective refractive index of the dominant mode can be represented as $n_{{{\text{eff}}}} = \beta /k_{0}$, subsequently, according to indexed waveguide theory, the phase imparted by each DSIN of specific diameter can be calculated in terms of effective refractive index as $\phi = \left( {2\pi /\lambda } \right) \cdot n_{{{\text{eff}}}}\cdot H ,$ where $\lambda$ represents the operational wavelength. Due to limited computational resources, meta-holograms consisting of an array of 175 × 175 pixels is numerically simulated and obtained results are presented in the above section.

## Conclusion

In conclusion, to fulfill the gap generated by the absence of the appropriate lossless dielectric material for near and deep ultraviolet wavelengths, we proposed diamond material as a best-suited candidate to demonstrate highly efficient phenomena. Based on the concept of index waveguide theory, a comprehensive analytical study (supported by the numerical simulations) of circular cylindrical diamond step-index nanowaveguides (DSINs) is presented. Finite-Difference Time-Domain technique based numerical simulations are performed for optimization of DSIN to achieve the efficient control of phase and amplitude of impinged ultraviolet light. To validate the proposed analytical modeling and to verify the acquired phase control along with maximum possible transmission amplitude, highly efficient polarization-insensitive meta-hologram is demonstrated for ultraviolet regime (particularly for an operational wavelength of ${\uplambda } =$ 250 nm). Holographic images “NUST” and “ITU” are reconstructed in the far-field region possessing sufficient high transmission efficiency and image fidelity. Due to limited computational resources, metasurface having an array of 175 × 175 elements is numerically simulated to demonstrate the meta-holograms.

## Data and material avability

All data required to aveluate the findings of this work is available in the presented paper. Addational data related to this work may be requested from the authors. All data and analysis details presented in this paper are avialable upon request to F.A.T.
